# Age-Related Changes in the Cochlea and Vestibule: Shared Patterns and Processes

**DOI:** 10.3389/fnins.2021.680856

**Published:** 2021-09-03

**Authors:** Vasiliki Paplou, Nick M. A. Schubert, Sonja J. Pyott

**Affiliations:** ^1^Department of Otorhinolaryngology and Head/Neck Surgery, University of Groningen, University Medical Center Groningen, Groningen, Netherlands; ^2^Research School of Behavioural and Cognitive Neurosciences, Graduate School of Medical Sciences, University of Groningen, Groningen, Netherlands

**Keywords:** auditory – visual perception, vestibular, inner ear, presbycusis or age-induced hearing loss, balance disorders, aging, inflammation, oxidative stress

## Abstract

Both age-related hearing loss (ARHL) and age-related loss in vestibular function (ARVL) are prevalent conditions with deleterious consequences on the health and quality of life. Age-related changes in the inner ear are key contributors to both conditions. The auditory and vestibular systems rely on a shared sensory organ – the inner ear – and, like other sensory organs, the inner ear is susceptible to the effects of aging. Despite involvement of the same sensory structure, ARHL and ARVL are often considered separately. Insight essential for the development of improved diagnostics and treatments for both ARHL and ARVL can be gained by careful examination of their shared and unique pathophysiology in the auditory and vestibular end organs of the inner ear. To this end, this review begins by comparing the prevalence patterns of ARHL and ARVL. Next, the normal and age-related changes in the structure and function of the auditory and vestibular end organs are compared. Then, the contributions of various molecular mechanisms, notably inflammaging, oxidative stress, and genetic factors, are evaluated as possible common culprits that interrelate pathophysiology in the cochlea and vestibular end organs as part of ARHL and ARVL. A careful comparison of these changes reveals that the patterns of pathophysiology show similarities but also differences both between the cochlea and vestibular end organs and among the vestibular end organs. Future progress will depend on the development and application of new research strategies and the integrated investigation of ARHL and ARVL using both clinical and animal models.

## Introduction

Aging causes the function of multiple organ systems to decline (Khan et al., 2017), and, like other sensory organs, the inner ear is also susceptible to the effects of aging. The inner ear is comprised of two sensory structures: the cochlea, which is responsible for the sense of hearing, and the vestibular end organs, which are responsible for the sense of balance (along with vision, proprioception, and motor systems). Both structures show age-related changes in structure and function, and both age-related hearing loss (ARHL), or presbycusis, and age-related loss of vestibular function (ARVL), also called presbyastasis or presbyequilibrium, are common among the older population. The personal, societal, and economic burden of ARHL and ARVL are substantial, whereas the prevention and treatment strategies are still quite limited. Moreover, both ARHL and ARVL can directly and indirectly exacerbate other effects of aging.

The cochlea provides the brain with acoustic information, which is essential for communication and personal safety. ARHL is typified by progressive, bilateral, symmetric hearing loss, especially in the high frequencies, and accompanied with difficulty understanding speech, especially in noise. ARHL is the most prevalent sensory deficit in the elderly (Bowl and Dawson, 2019) and is associated with an increased odds of social isolation (Mick et al., 2014), an increased odds of reported falling (Lin and Ferrucci, 2012), accelerated cognitive decline and incident cognitive impairment (Lin et al., 2013), and an increased risk of dementia (Lin et al., 2011a). Moreover, hearing aid use is associated with reduced odds of depression (Mener et al., 2013). Importantly, these studies indicate links but do not distinguish common from causal mechanisms. In addition to significant health burden and reduction in the quality of life (Ciorba et al., 2012), ARHL results in substantial economic burden in both direct medical costs and costs attributable to lost productivity (Stucky et al., 2010). Unlike conductive hearing loss, which can often be treated, restoration of hearing in cases of sensorineural hearing loss, by far the most common cause of ARHL, is not possible. Hearing aids that amplify sounds and cochlear implants, which bypass the sensory transduction machinery of the inner ear and directly stimulate the auditory nerve, can be used to mitigate symptoms but do not fully restore auditory input.

The end organs of the vestibular system provide the brain with information necessary for detection of self-motion, spatial orientation, and postural control (Liston et al., 2014). ARVL can present as dizziness but vestibular hypofunction can also go undetected (van de Berg et al., 2015). Regardless of presentation, dizziness, imbalance, or even simply feeling unsteady diminishes the quality of life of older adults (Agrawal et al., 2018). The declining ability to perform daily tasks, the loss of independence, and the fear of falling can lead to anxiety and depression. ARVL contributes to the increased likelihood and severity of falls (Herdman et al., 2000), which are the leading cause of fatal and trauma-related injuries among older adults (Scuffham et al., 2003; Stevens et al., 2006). Moreover, balance disorders were recently associated with all causes of mortality in older adults (Cao et al., 2021). Finally, recent work indicates that ARVL is associated with the increased likelihood and rate of cognitive decline in the aging (Semenov et al., 2016; Dobbels et al., 2019). These studies indicate links between balance disorders, which involve multiple sensory and motor systems, and do not identify shared or causal mechanisms. Not surprisingly, the estimated economic burden of vestibular disorders is enormous (Sun et al., 2014; Kovacs et al., 2019). Unfortunately, both the clinical detection and management of ARVL is limited (Anson and Jeka, 2015; Arshad and Seemungal, 2016; Renga, 2019).

Like other multifactorial conditions, ARHL and ARVL show substantial individual variation and result from a combination of both intrinsic factors, such as genetics, as well as external factors, like environmental conditions (e.g., noise exposure), lifestyle factors (e.g., smoking), medications (e.g., ototoxic drugs), and other comorbidities (e.g., cardiovascular disease). ARHL (recently reviewed in Bowl and Dawson, 2019; Fischer et al., 2020; Keithley, 2020; Tawfik et al., 2020; Wang and Puel, 2020) and ARVL (reviewed in Sturnieks et al., 2008; Anson and Jeka, 2015; Fernandez et al., 2015; Iwasaki and Yamasoba, 2015; Arshad and Seemungal, 2016; Brosel et al., 2016; Ji and Zhai, 2018; Brosel and Strupp, 2019; Jahn, 2019) are often investigated separately even though both conditions rely on age-related changes in the same sensory structure. Therefore, insight essential for the development of treatments for ARHL and ARVL can be gained by careful examination of their shared and unique pathophysiology in the inner ear. To this end, the review begins by comparing the prevalence patterns of ARHL and ARVL. Next, the normal structure and function of the auditory and vestibular end organs and their age-related changes are compared. Then, the contributions of various molecular mechanisms, notably inflammaging, oxidative stress, and genetic factors, are evaluated as possible common culprits that interrelate age-related pathology in the cochlea and vestibule as part of ARHL and ARVL. The links between age-related changes in the peripheral and central auditory and vestibular systems are then highlighted. Finally, future directions to advance investigation of ARHL and ARVL are offered.

## Prevalence of ARHL and ARVL

**FIGURE 1:**
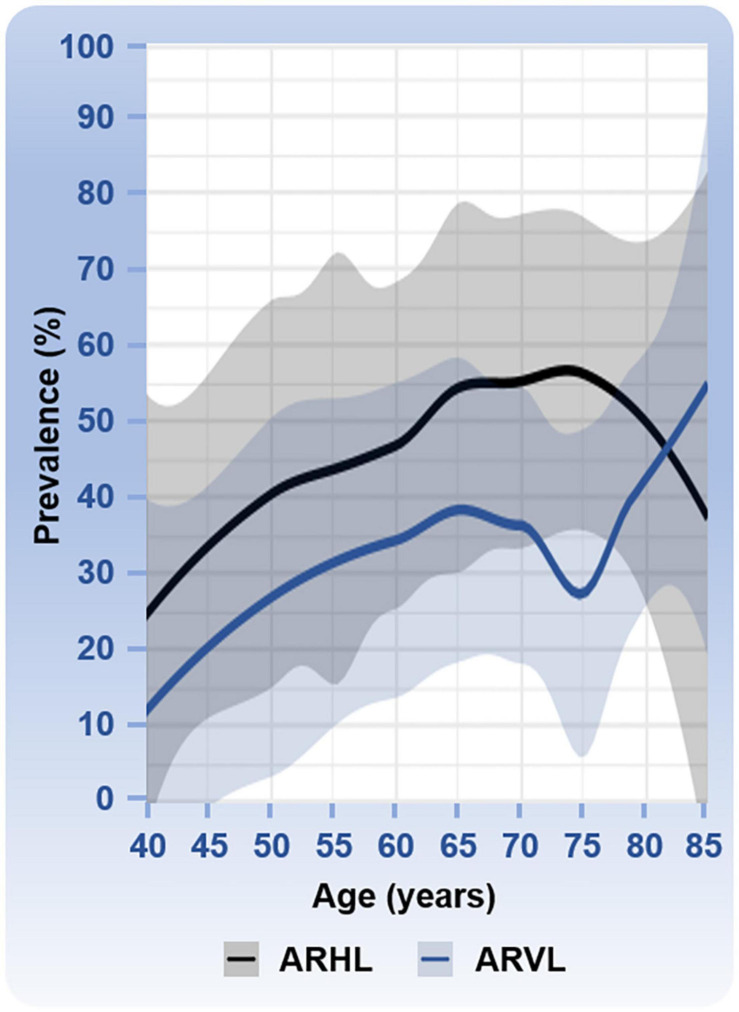
Prevalence of age-related hearing loss (ARHL) and age-related vestibular loss (ARVL). Prevalence numbers (age range from 40 to 85 years) are based on six studies with various sample sizes for ARHL: *N* = 654,113 (Hogan et al., 2009), *N* = 3,831 (Hoffman et al., 2017), *N* = 3,184 (Feder et al., 2015), *N* = 4,357 (Gong et al., 2018), *N* = 5,742 (Agrawal et al., 2008), and *N* = 7,490, (Lin et al., 2011b); and four studies with various sample sizes for ARVL: *N* = 3,197 (Jönsson et al., 2004), *N* = 6,785 (Agrawal et al., 2009), *N* = 216 (Tan et al., 2016), and *N* = 1,000 (Colledge et al., 1994). Mean percentages with the 95% confidence intervals are shown. ARHL was defined as followed in the different studies: a hearing disability associated with communication difficulty and/or use of hearing aids (Hogan et al., 2009); high-frequency hearing loss (Hoffman et al., 2017); combined mild, moderate, or worse hearing loss in high frequencies (Feder et al., 2015); hearing loss ≥25 dB HL (Gong et al., 2018); high frequency hearing loss overall (Agrawal et al., 2008); and bilateral hearing loss ≥25 dB HL (Lin et al., 2011b). ARVL was determined based on reported overall occurrence of balance symptoms (Jönsson et al., 2004), the Romberg testing and difficulty with balance or falling in the past 12 months (Agrawal et al., 2009), the clinical test for sensory interaction on balance (Tan et al., 2016), and a dizziness questionnaire (Colledge et al., 1994). The clinical test of sensory interaction and balance provides information about the ability to stand upright under six sensory conditions: (1) quiet standing on the floor, looking straight ahead; (2) quiet standing on the floor with eyes closed; (3) quiet standing on the floor wearing the conflict dome; (4) quiet standing on the foam, with eyes open; (5) quiet standing on the foam, with eyes close; and (6) quiet standing on the foam wearing the conflict dome (Cohen et al., 1993). One study reported an especially low prevalence of ARVL at 75 years of age (Tan et al., 2016). One study cited in the text was not used in this analysis because the study included only two age categories, 70 to 80 years and >80 years (Zuniga et al., 2012).

Estimates of the prevalence of ARHL and associated risk factors have been conducted using large cohorts. Prevalence is the number of individuals with a given condition in a given population at a specific point in time. Although the patterns of ARHL vary, several studies have used well-defined criteria, and a summary of the prevalence numbers from these studies is shown in Figure 1. Epidemiological studies also identified the risk factors associated with ARHL (reviewed in Yamasoba et al., 2013). Males are more likely to have ARHL. Modifiable risk factors associated with the increased likelihood of ARHL include greater exposure to noise, smoking, and comorbidities of hypertension and cardiovascular disease, cerebrovascular disease, and diabetes. In contrast to ARHL, the prevalence of ARVL has been less investigated. An often-cited study using the modified Romberg Test of Standing Balance on Firm and Compliant Support Surfaces reported an overall prevalence of vestibular dysfunction in just over one-third of adults aged 40 years and older in the United States (Agrawal et al., 2009). In this study and a recent follow up study (Cao et al., 2021), the odds of vestibular dysfunction were higher in individuals with lower educational status, lower physical activity, and with diabetes or cardiovascular disease. In this study, the odds of vestibular dysfunction were lower in individuals with greater education and higher in those with diabetes. Other studies have assessed the prevalence of ARVL using the occurrence of symptoms such as vertigo, dizziness, and dysequilibrium (Colledge et al., 1994; Jönsson et al., 2004). A few, smaller sized studies have used clinical assessments of both auditory and vestibular function (Zuniga et al., 2012; Tan et al., 2016). A summary of the prevalence numbers from most of these studies investigating ARVL is also shown in Figure 1.

When comparing the prevalence patterns between ARHL and ARVL (Figure 1), both rise most steeply between the ages of 40 and 60 to 70 years of age. Interestingly the prevalence of ARHL decreases slightly after the age of 75 years whereas ARVL continues to increase with age. The apparent dip in prevalence of ARVL at 75 years of age arises from one study that reported a considerably lower prevalence at this age (Tan et al., 2016) compared to other studies. This dip in ARVL prevalence around 75 years of age should be interpreted cautiously considering that studies included were cross-sectional, assessed vestibular dysfunction with different methods (e.g., questionnaires versus balance testing), and utilized different age inclusion criteria such that not all studies cover the complete age range. Longitudinal studies would be necessary to examine trends over time. Moreover, the various ways in which vestibular dysfunction is measured in these studies complicates interpretation and likely underestimates the overall prevalence of ARVL. Specifically, hypofunction of the vestibular end organs is not always associated with dizziness (van de Berg et al., 2015) and, therefore, could have been missed in some of these studies. Importantly, the incidence, or occurrence of new cases within a given period of time, of ARHL and ARVL is also expected to increase due to the rapidly increasing aging population. ARHL is estimated to affect 60 million Americans above the age of 64 by the year 2025, an increase in prevalence from 9.3% in 2007 to 19% of the total population in 2025 (Liu and Yan, 2007), and ARVL is expected to increase from 12.9% in 2015 to 23.7% in 2050 (Zalewski, 2015).

## Anatomy and Functional Assessment of the Cochlea and Vestibular End Organs

**FIGURE 2:**
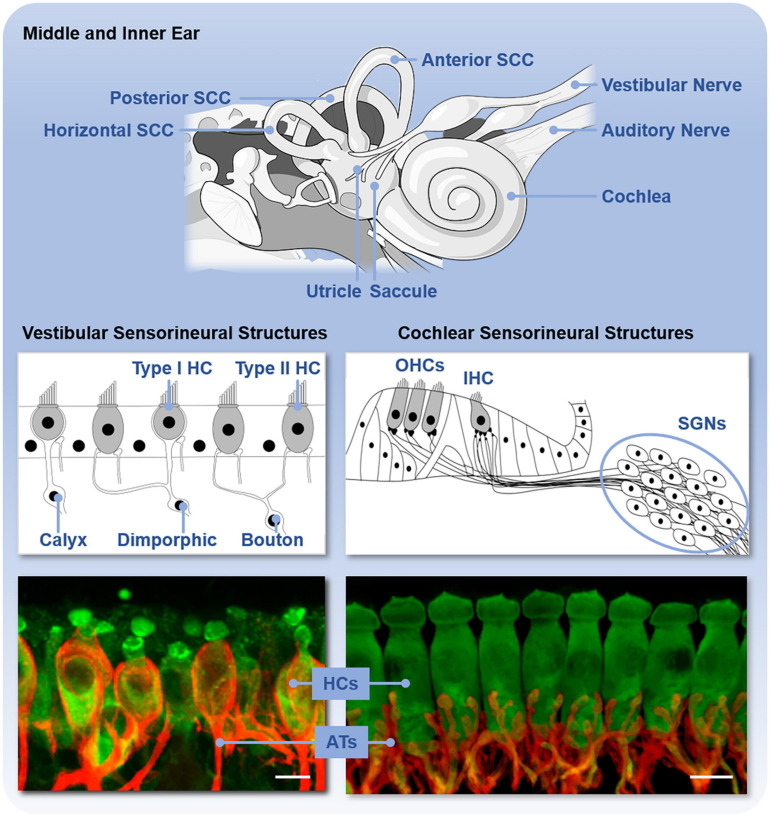
Anatomy of the inner ear and sensorineural structures. The upper panel shows the inner ear with vestibular end organs (including the utricle, saccule, and three semicircular canals, SCC) and cochlea labeled. The middle panel shows the schematic organization and types of sensory hair cells (HC) and primary neurons in the vestibular end organs and cochlea. The vestibular end organs contain type I and type II hair cells (HC) and vestibular neurons with either calyx, dimorphic, or bouton afferent terminals. The cochlea contains outer hair cells (OHCs) and inner hair cells (IHCs) and spiral ganglion neurons (SGNs). The bottom panel shows these sensorineural structures isolated from C57BL/6 mice and immunofluorescently labeled to reveal the hair cells (HCs, green) and primary afferent terminals (ATs, red). Scale bar is 10 μm.

The inner ear consists of both the auditory and vestibular end organs (Figure 2). The auditory and vestibular structures of the inner ear share the same embryonic origin (Morsli et al., 1998) and utilize similar yet distinct sensorineural structures and mechanisms of transduction. The auditory end organ consists of a coiled tube called the cochlea. Projections from the primary sensory neurons, with cell bodies housed in the spiral ganglion, are relayed first to the cochlear nucleus in the brainstem and ultimately reach the primary auditory cortex for the conscious perception of sound (reviewed in Pickles, 2015). The vestibular end organs consist of two otolithic organs – the utricle and saccule, which detect linear acceleration in the horizontal and vertical planes, respectively, – and the horizontal, anterior, and posterior semicircular canals, which detect head angular accelerations around the vertical, sagittal, and frontal axes. The otolithic structures are so named because of the otoconia, calcium carbonate structures, that lie above the hair cells. Sensory signals from the vestibular end organs are relayed via the primary vestibular neurons, with cell bodies housed in Scarpa’s ganglion, to the vestibular nuclei in the brainstem as well as the cerebellum. Projections then connect to the reticular formation, the spinal cord, and the thalamus. Vestibular signals contribute to spatial perception and orientation as well as automatic reflexes and motor coordination (reviewed in Kingma and van de Berg, 2016).

The sensory hair cells and peripheral dendrites of the primary sensory neurons are housed within the neuroepithelia that are, in turn, housed in the bony encasements of these end organs. The classes of sensory hair cells and primary neurons and their arrangements in the sensory epithelium vary between the end organs (Figure 2). In the cochlea, there are two types of sensory auditory hair cells – inner and outer hair cells – and two types of primary auditory neurons – type I and II spiral ganglion neurons. These hair cells are arranged tonotopically along the length of the neuroepithelium, called the organ of Corti. Vestibular end organs contain two types of hair cells – type I and II hair cells – and three types of primary vestibular neurons – calyx, dimorphic, and bouton neurons – distinguished by the morphology of their afferent terminals. The distribution and relative abundance of the distinct types of hair cells and vestibular neurons varies spatially across the vestibular neuroepithelia. Moreover, within the vestibular neuroepithelia, there are zonal differences in the spike generation patterns – regular and irregular – of the vestibular neurons (Goldberg, 2000). Both the organ of Corti and vestibular neuroepithelia also receive efferent innervation from the brain that modifies afferent input in yet incompletely understood ways. In the mature organ of Corti, medial olivocochlear efferents originate in the medial superior olive and synapse directly on the outer hair cells. Lateral olivocochlear efferents originate in the lateral superior olive and terminate on the dendrites of type I auditory nerve afferent fibers beneath inner hair cells (Lopez-Poveda, 2018). Within vestibular neuroepithelia, efferent innervation arises from the parvocellular reticular nucleus (Warr, 1975) and synapses onto type II hair cells as well as both calyx and bouton afferent terminals (Lysakowski and Goldberg, 1997, 2008; Jordan et al., 2015). Regardless of hair cell type, deflection of the hair bundle, due to either sound or head movement, opens mechanotransduction channels in the hair bundles that leads to depolarization of the hair cells, release of neurotransmitter, and activation of the primary auditory or vestibular neurons. These mechanisms have been comprehensively reviewed elsewhere (e.g., Fettiplace, 2017).

The hair cells in both the cochlear and vestibular end organs are bathed in endolymph, which, in contrast to many other extracellular fluids, has a relatively high concentration of potassium ions (K^+^) and low concentration of sodium ions (Na^+^). This concentration gradient means that influx of K^+^ is depolarizing (reviewed in Hibino and Kurachi, 2006; Ciuman, 2009; Nin et al., 2016; Köppl et al., 2018). The ionic composition of the endolymph gives rise to the endolymphatic potential, which provides the driving force for hair cell mechanotransduction. In the cochlea, the endolymph is generated by the stria vascularis in the lateral wall. Much less is known about the endolymph-generating structures in the vestibular end organs, although there are probably distinct structures in each of the five end organs. Measurements indicate that the endolymphatic potential is substantially higher in the cochlea (+80 to +120 mV) than the vestibular end organs (+1 to +11 mV), but accurate measurements of the EP, especially in the microenvironment bathing the hair cells, is difficult. Direct measurement of endolymphatic potential is possible in animal models but not possible in healthy humans.

A variety of clinical assessments of peripheral auditory function in humans are available and include more commonly pure tone audiometry (PTA) and less commonly measurements of auditory brainstem responses (ABRs) (Hoth and Baljic, 2017). Similar assessments to assess cochlear function are available in animal models, although, in contrast to humans, measurement of ABRs (e.g., Willott, 2006) are more easily and, therefore, more commonly performed compared to behaviorally measured audiograms (e.g., Heffner et al., 2001). Importantly these two measures produce different absolute thresholds, with behaviorally measured thresholds often lower than electrophysiologically measured thresholds but estimations of induced hearing loss (absolute threshold shifts) being comparable in animal models (e.g., Borg and Engstrom, 1983). Objective clinical assessments of peripheral vestibular function most commonly include (van de Berg et al., 2018): (1) measurement of the cervical vestibular evoked myogenic potentials (cVEMP), which indicates the status of the saccule; (2) the ocular VEMP (oVEMP), which indicate the status of the utricle; (3) the caloric test, which indicates status of the horizontal semicircular canal; and (4) the head impulse test (HIT), which indicates status of the horizontal or anterior and posterior semicircular canals. The HIT involves an abrupt, high-acceleration, small amplitude rotation of the head by the examiner while the subject looks at a target straight ahead (Agrawal et al., 2014). To maintain gaze stability, the normal vestibulo-ocular reflex (VOR) produces an eye movement nearly equal in velocity and only slightly delayed in time in the opposite direction of head movement. The VOR response elicited by the HIT can be measured using video oculography (vHIT). Because of practical limitations imposed by video oculography, vHIT requires the examiner to apply head impulses to the subject from the back of the head and used to measure function of the horizontal canals only (Macdougall et al., 2013). Compared with HIT, vHIT has a higher sensitivity and specificity and can help differentiate between central and peripheral vertigo in patients with acute vestibular syndrome (Bartl et al., 2009; MacDougall et al., 2009). In addition to the cVEMP, oVEMP, and HIT, a variety of other screening tests are also available (reviewed in Cohen, 2019).

In animal models, peripheral vestibular function is often assessed by examination of reflex responses (e.g., Martins-Lopes et al., 2019) or measurements of vestibular evoked potentials (VsEPs), an electrophysiologic response to a change in linear acceleration (i.e., jerk) of the head that can be performed in animals with relatively small heads, such as rodents and birds (e.g., Jones et al., 2011). Moreover, in both humans and animal models, measurements of peripheral vestibular function are difficult owing to the small electrical potentials generated by the end organs and the widespread and diffuse innervation of the primary neurons in the brain. As a result, methods of assessment are often indirect and detect or are influenced by myogenic, proprioceptive, or visual responses (reviewed in Llorens et al., 2018). For example, cVEMPs are inhibitory potentials and depend on the magnitude of the contraction of the sternocleidomastoid muscles measured by electromyography (EMG). Thus, age-related loss of muscle mass and strength (sarcopenia) can also reduce the magnitude of cVEMP responses and complicate interpretation of age-related changes in vestibular function specifically. In comparison to peripheral auditory function, analogous assessments of peripheral vestibular function in humans and animal models are more limited. The development of analogous and robust assessments of peripheral vestibular function in humans and animal models is sorely needed to progress vestibular research.

## Age-Related Changes in the Anatomy and Function of the Cochlea and Vestibular End Organs

**FIGURE 3:**
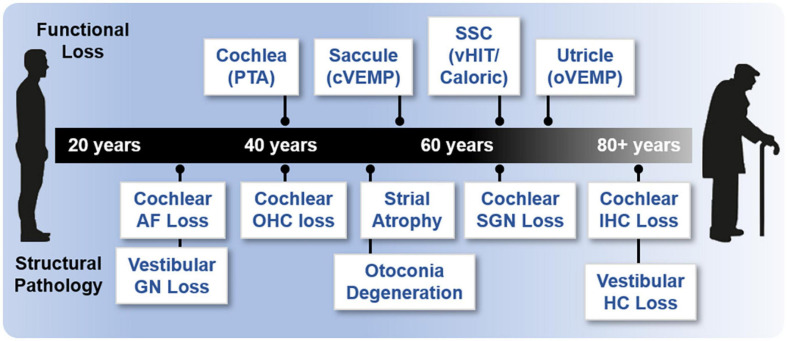
Onset of age-related functional and structural changes in the inner ear. Age of onset of functional loss in humans is based on various methodologies and references. Loss of cochlear function is based on pure tone audiometry (PTA; 40 years) (Parthasarathy et al., 2020). Saccular function (55 years) is based on cVEMP; semicircular canal (SSC) and horizontal canal (HC) function (65 years) are based on vHIT and caloric measurements; and utricular function (70 years) is based on oVEMP (Zapala et al., 2008; Maes et al., 2010; Ji and Zhai, 2018). Various references were used to assess structural histopathology. Loss of cochlear auditory nerve (AN) fibers (>20% loss, 30 years), loss of cochlear outer hair cells (OHCs, >20% loss, 40 years), and loss of cochlear inner hair cells (IHCs, >20% loss, 80 years) are derived from Wu et al. (2019). Loss of cochlear spiral ganglion neurons (SGN, >20% loss, 35 years) are derived from the regression in Makary et al. (2011). Cochlear strial atrophy (>20% loss in apical turns) is derived from Suzuki et al. (2006). Loss of vestibular ganglion neurons (GN, age of onset, 30 years) is reviewed in two studies (Zalewski, 2015; Ji and Zhai, 2018). Otoconia degeneration (50 years) is reviewed previously (Walther and Westhofen, 2007). Loss of vestibular hair cells (HC, all structures, age of onset) is reviewed in two studies (Zalewski, 2015; Ji and Zhai, 2018) but there is a large discrepancy (see also Merchant et al., 2000; Walther and Westhofen, 2007).

Various studies have used functional assessments to characterize ARHL and ARVL (Figure 3). These studies have shown that age-related hearing loss assessed by pure-tone audiograms begins at 40 years of age and progresses bilaterally from high to low frequencies (summarized in Parthasarathy et al., 2020). The rate of hearing decline with age is highly variable (Gates and Cooper, 1991). Although there are fewer studies quantitatively assessing ARVL, patterns are beginning to emerge (reviewed in Zalewski, 2015; Ji and Zhai, 2018). Decrease in saccular function (indicated by cVEMP amplitudes) begins around the age of 50 to 60 years. A decrease in utricular function (indicated by oVEMP amplitudes) has also been reported after the age of 60 to 80 years. Thus, age-related decline in saccular and utricular function is consistently reported. In contrast, inconsistent findings are reported for horizontal canal function (indicated by the caloric test), with some studies reporting loss of function after the age of 66 years (Maes et al., 2010) and others reporting no age-related changes in function (Mallinson and Longridge, 2004; Zapala et al., 2008). Assessments of the semicircular canals by vHIT indicated age-related decreases in function do not begin until 80 years of age (reviewed in Ji and Zhai, 2018). Collectively, these findings indicate that the otolithic structures in comparison to the semicircular canals are more vulnerable to aging. Again, these assessments, are potentially confounded by the contribution of central compensation and/or aging of the motor and sensory pathways rather than or in addition to the vestibular end organs. Moreover, there is considerable individual variation, reflected in the large age-related normative values in these measures (Zalewski, 2015). Finally, a combination of various tests, which provide complementary rather than redundant information, are necessary for completely assessing ARVL (Krager, 2018).

Together and despite the caveats highlighted above, the functional studies available indicate that in humans ARHL precedes ARVL. In many regards, studies directly comparing peripheral ARHL and ARVL using equivalent assessments of evoked potentials (ABR and VsEP) in various strains of inbred mice show similar findings to observations in humans. Specifically, age-related decline in cochlear function generally precedes decline of vestibular function (Jones et al., 2006), and there is considerable variation in the age-related decline in both cochlear (Zheng et al., 1999) and peripheral vestibular (Jones et al., 2006) function among individuals of the same strain and across strains. Given the differences in the dynamic range when comparing ABR- and VsEP-based assessments of the cochlea and vestibular end organ function (approximately 60 dB for ABR and 15 dB for VsEP), direct comparisons in these measurements and interpretation of their changes with age should be made cautiously. There is limited investigation of the correlation between clinical assessments of age-related decline of auditory and vestibular function within the same subjects. One study reported a significant correlation between ARHL (assessed with PTA) and reduced saccular function (assessed by cVEMP amplitudes) but not utricular function (assessed with oVEMP amplitudes) (Zuniga et al., 2012). Another association study reported no correlation between ARHL and ARVL (assessed using a clinical test of sensory interaction on balance) (Tan et al., 2016). More comparisons using direct and analogous assessments of inner ear function in the same individuals are necessary, especially given the large range of normative values of these measures.

Turning attention to age-related structural changes in the in the inner ear (Figure 3), a variety of human histological studies documented age-related hair cell loss, spiral ganglion neuron loss, and atrophy of the stria vascularis (reviewed in Merchant and Nadol, 2010). The various forms of histopathology have been linked to patterns of hearing loss indicated by audiometric testing (PTA) and led Schuknecht and colleagues to classify four main types of presbycusis: sensory, neural, metabolic or strial, and cochlear conductive (Schuknecht, 1964; Schuknecht and Gacek, 1993). More recent human histological studies documented the loss of afferent fibers and synapses between the inner hair cells and type I afferent fibers (Viana et al., 2015; Wu et al., 2019). The most recent study examined normally aging ears and used quantitative microscopic analysis and subsequent statistical modeling to relate loss of hair cells and auditory nerve fibers as well as atrophy of strial tissues to ARHL indicated by the audiogram (Wu P. Z. et al., 2020). In contrast to previous predictions based on histopathological findings, this study indicates that ARHL is well predicted by outer and then inner hair cell loss and that, perhaps unexpectedly, strial atrophy (although commonly observed) and nerve fiber loss contribute inconsequentially to ARHL. This recent study included cochleae examined previously (Schuknecht and Gacek, 1993). Thus, differences in this most recent study arise from the (1) larger number of specimens investigated, (2) quantification of fractional (and not complete) hair cell loss, and (3) statistical methodology employed. When linking structural and functional pathology, this study suggests that loss of cochlear amplification is the key contributor to ARHL in humans. Importantly, the effects of noise-induced hearing loss, which may be substantial, cannot be disentangled from the effects of ARHL. Moreover, the histological methods used do not reveal structural or functional pathology completely. For example, decline in strial function probably occurs before quantifiable strial atrophy. A variety of animal models have been used to identify histopathological changes associated with loss of cochlear function as part ARHL (reviewed in Kujawa and Liberman, 2019). Depending on the model, ARHL has been correlated with either hair cell loss, strial degeneration, or afferent synapse/fiber loss. Thus, across animal models there does not seem to be a single histopathology underlying ARHL. In these studies, animals are raised in controlled environments, and, unlike humans, show ARHL that has been largely spared from exposure to noise (but see Lauer et al., 2009) or other ototoxins. Thus, differences across animal models may better reflect different genetically determined patterns in the histopathology underlying ARHL.

Structural changes to the vestibular system with increasing age have been investigated in various human histological studies (reviewed in Zalewski, 2015; Ji and Zhai, 2018) but their relation to ARVL is not defined. When considering the sensory hair cells, some studies detected a decrease in the number of both type I and type II hair cells in different regions of the five vestibular end organs in subjects with a mean age of 84 and 94 years (Merchant et al., 2000; Rauch et al., 2001; Lopez et al., 2005; Walther and Westhofen, 2007). In contrast, another study showed no age-related loss of hair cells in the utricles of subjects with a mean age of 82 years (Gopen et al., 2003). When considering the afferent neurons, separate studies showed that the number of cells in Scarpa’s ganglion steadily decreases after the age of 30 years (Velazquez-Villasenor et al., 2000; Park et al., 2001). Another earlier study showed that, while there was no decline in the number of afferent fibers during aging, deposition of amyloid bodies could be detected (Gopen et al., 2003). Loss of synapses between the hair cells and afferent fibers has not been investigated in human vestibular end organs, although recent work in FVB/N mice documented age-related loss of afferent synapses in discrete regions of the utricle (specifically calyceal innervations in the utricular extrastriolar region) (Wan et al., 2019). Direct comparison of peripheral (cholinergic) efferent innervation in the gerbil revealed significant loss of olivocochlear (OC) neurons but no change in efferent neurons of the vestibular system in aged compared to young adult animals (Radtke-Schuller et al., 2015). Finally, otoconia in both the otolithic structures show decreases in numbers and volumes (Igarashi et al., 1993; Walther and Westhofen, 2007; Walther et al., 2014), with otoconia in the saccule showing worse age-related loss in volume (Igarashi et al., 1993).

Importantly, direct comparison of structural pathology with vestibular function is not available from these studies. Nevertheless, a decline in the number or morphology of vestibular ganglion cells is consistent with decreased neurotransmission and declining vestibular function. Greater loss of otoconia in the saccule compared to utricle is consistent with earlier declining function of the saccule compared to utricle. In general, loss of otoconia may explain the earlier decline in function of the otolithic structures compared to the semicircular canals. Finally, no studies have investigated the age-related integrity of afferent synapses in the human vestibular end organs. However, intriguing earlier work showed that asymptomatic vestibular disorders (revealed by abnormal caloric test results) are common in patients with auditory neuropathy when a peripheral neuropathy is also present (Fujikawa and Starr, 2000). The link between age-related structural and functional changes in the vestibular end organs is also still unclear from animal models. In C57BL/6 mice, vestibular hair cells in the lateral semicircular canals were significantly reduced in 24-week-old mice whereas VOR gain (a measure of semicircular canal function) was significantly reduced only in the oldest (60-week-old) mice (Shiga et al., 2005). In contrast, another study reported age-related elevations in VsEP thresholds that were correlated with the loss of utricular ribbon synapses but not hair cells or neurons (Wan et al., 2019). These studies indicate the potential confounds of central compensation when examining vestibular function in animal models (e.g., Cassel et al., 2019) and the need to examine multiple structures and use sensitive functional assessments. Moreover, experiments utilizing C57BL/6 mice must always be interpreted cautiously considering this strain shows accelerated age-related hearing loss (Zheng et al., 1999).

## Mechanisms Underlying Age-Related Pathology in the Cochlea and Vestibular End Organs as Part of ARHL and ARVL

### Inflammaging

Inflammation is a hallmark of aging, and other coexisting comorbidities linked to aging, such as cardiovascular disease, type 2 diabetes, and Alzheimer’s (Blasko et al., 2004; Ferrucci and Fabbri, 2018). Inflammation is also associated with ARHL (Fujioka et al., 2014). Inflammaging, a portmanteau of inflammation and aging, is defined as chronic, low-grade inflammation that worsens with age and contributes to the pathogenesis of various age-related pathologies (reviewed in Baylis et al., 2013; Franceschi et al., 2018). Inflammaging arises from immunosenescence or cellular senescence of the immune system (reviewed in Muller et al., 2019). As immune system function diminishes with age, there is decreased ability to control or down-regulate the production of pro-inflammatory proteins. This slow and continuous build-up of pro-inflammatory proteins disrupts normal function of multiple systems. Thus, inflammaging seems to promotes inflammation, immunosenescence, and cellular senescence (Rodrigues et al., 2021). Importantly, inflammaging may not necessarily be a normal or inevitable part of the aging process but rather indicative of accelerated aging (Franceschi et al., 2018), suggesting that monitoring and mitigating inflammaging may have diagnostic and treatment value.

Although inflammatory and immune responses have been characterized in various forms of acquired hearing loss (Hu B. H. et al., 2018), attention has more recently focused on the role of inflammaging in ARHL (Watson et al., 2017). In humans, Verschuur and colleagues documented a significant association between four systemic markers of inflammation, white blood cell count, neutrophil count, interleukin 6 (IL-6), and C-reactive protein, and decreased hearing thresholds (Verschuur et al., 2012). These same researchers confirmed increased white blood cell counts with presbycusis in an independent population (Verschuur et al., 2014). Supporting a role for inflammaging, or at least aberrant immune cell activity, examination of human cochlear tissue using immunohistochemistry with confocal and super-resolution imaging revealed age-related changes in both the number and morphology of macrophages (Noble et al., 2019). Activated macrophages were more abundant in basal turns and in older cochleae and found in the lateral wall and auditory nerve. Evidence supporting a role of inflammaging in ARHL also comes from animal studies. Resident macrophages present in the basilar membrane from the basal region of the cochlea of C57BL/6J mice show morphological signs consistent with activation in cochleae from aging mice (up to 10 to 12 months old) (Frye et al., 2017). Recent transcriptomic investigation of young (6-week-old) and old (one-year-old) C57BL/6 mice, showed increased enrichment of genes associated with immune responses and inflammatory pathways in older cochleae (Su et al., 2020). Finally, the cochlea – although once believed to be immune privileged – is likely susceptible to systemic inflammation (Kampfe Nordstrom et al., 2018).

The involvement of inflammaging in ARVL has not been investigated. Nevertheless, the molecular players required for inflammaging are present in the vestibular end organs, although their association with ARVL requires further investigation. Macrophages and microglia have been identified immunohistologically in the tissue underlying the saccule, utricle, and all three ampullae of the semicircular canals, among Scarpa’s ganglion cells, and within the endolymphatic duct and sac in human adults with no history of hearing or balance disorders (O’Malley et al., 2016). Studies in adult C57BL/6J mice (aged 4 to 6 weeks) showed that local induction of inflammation caused significantly increased permeability and leakage of the blood-labyrinth barrier due to activation of perivascular-resident macrophage-like melanocytes (PVM/M) (Zhang et al., 2013). A similar role of PVM/M-mediated leakage of the blood-labyrinth barrier was linked to elevated high frequency hearing thresholds in this same strain of mice (Neng et al., 2015). Thus, increased permeability of the blood-labyrinth barrier may increase the susceptibility of the inner ear to systemic inflammatory processes.

**FIGURE 4:**
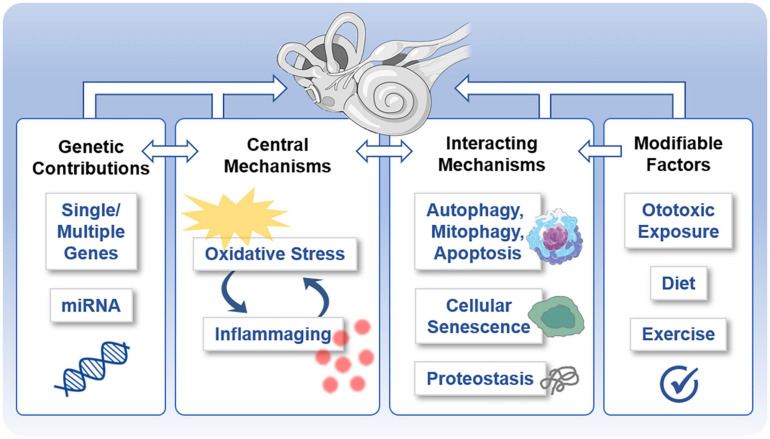
Potentially shared mechanisms underlying both age-related hearing loss (ARHL) and age-related vestibular loss (ARVL). Various molecular mechanisms underlie and interrelate age-related damage to the inner ear that gives rise to ARHL and ARVL. These include the central mechanisms of oxidative stress and inflammation, genetic contributions, interacting mechanisms, and potentially modifiable factors.

More work is needed to investigate the role of inflammaging in the cochlea and especially vestibular end organs (Figure 4). Because inflammaging appears to involve similar molecular players and mechanisms across diseases (Michaud et al., 2013), priority should be given to investigating the relationship of specific pro-inflammatory markers, such as IL-6, IL-1, CRP, and TNF-α, to morphological and functional measures of ARHL and ARVL. For these investigations, animal models are especially tractable. Likewise, age-related associations between systemic markers of inflammaging and vestibular function should be assessed in humans. Finally, a careful comparison of the patterns of inflammaging between inner ear end organs is necessary to reveal similarities and differences in mechanisms.

### Age-Related Oxidative Stress

Oxidative stress is implicated in various age-related conditions, such as cardiovascular diseases, chronic obstructive pulmonary disease, chronic kidney disease, neurodegenerative diseases, and cancer (reviewed in Liguori et al., 2018), including ARHL (reviewed in Seidman et al., 2004; Henderson et al., 2006; Prasad and Bondy, 2020) and ARVL (see Iwasaki and Yamasoba, 2015; Brosel et al., 2016). In this model (reviewed in Luo et al., 2020), age-related damage results from accumulated oxidative damage caused by free radicals known as reactive oxygen species (ROS). ROS are a natural byproduct of cellular respiration occurring in the mitochondria and play important roles in cell signaling and homeostasis (Zorov et al., 2014). Environmental stressors can also result in abnormal ROS production (Pizzino et al., 2017). Commonly defined ROS include superoxide radicals, hydrogen peroxide, hydroxyl radicals, and singlet oxygen (Valko et al., 2007). ROS may act either as free radicals directly or are readily capable of generating free radicals. ROS and free radicals can damage DNA, break down lipid and protein molecules, and trigger cell death, all of which contribute to damage to cochlear structures, specifically hair cells. Various antioxidant systems are in place to clear ROS (Valko et al., 2007). These include enzymes involved in glutathione (GSH) metabolism, such as glutathione S-transferase (GST), glutathione peroxidase (GPX), and glutathione reductase (GSR). Furthermore, enzymes involved in superoxide anion and hydrogen peroxide breakdown, catalase (CAT) and superoxide dismutase (SOD), respectively, are also crucial for the clearance of ROS. Another enzyme, methionine sulfoxide reductase (MSR), is important in reducing methionine residues that have been oxidized by ROS. Oxidative stress results from the dysregulation of redox homeostasis, with a relative increase in ROS and decrease in antioxidant capacity.

In humans, several studies identified significant associations between ARHL and polymorphisms in enzymes that have antioxidant-related roles in the cochlea, including CYP1A1, NAT2, GSTM1 and the GSTT1, UCP2 (Unal et al., 2005; Van Eyken et al., 2007; Bared et al., 2010; Angeli et al., 2012; Manche et al., 2016; Karimian et al., 2020). The link between oxidative stress and ARHL has prompted multiple investigations into the effectiveness of antioxidants in the prevention of ARHL, but the findings are inconclusive (reviewed in Tavanai and Mohammadkhani, 2017). Much less work has been done to examine the contribution of oxidative stress to ARVL, but a recent study showed that biomarkers of oxidative stress are significantly elevated in patients with chronic subjective dizziness (Fang et al., 2020).

In animal models, several studies implicate oxidative stress in the initiation and progression of damage to the inner ear due to various insults. Although fewer studies have examined the contribution of oxidative stress to both ARHL and ARVL, the existing work suggests that the cochlea and vestibular end organs utilize overlapping but also distinct antioxidant enzymes and that these structures show differences in the age-related changes in these antioxidant enzymes. In rat, reverse transcription PCR and immunohistochemical staining revealed distinct expression of specific members of the MSR family of proteins between the cochlea and vestibular end organs as well as among cell types within the vestibular end organs (Kwon et al., 2014). Different patterns in age-related changes in antioxidant enzymes have also been documented in rat (Coling et al., 2009). In this study, a significant increase in CAT activity was observed in vestibular end organs but not the cochlea in old (24-month-old) compared to young (3-month-old) rats. In contrast, age-related increase in GPX activity was observed in the cochlea but not the vestibular end organs. Finally, redox state (measured by the ratio of reduced compared to oxidized glutathione) was increased in vestibular end organs but decreased in the cochlea tissue. In separate studies, mice lacking superoxide dismutase 1 (SOD1) show accelerated age-related loss of cochlear hair cells (Keithley et al., 2005) but not vestibular hair cells (Johnson et al., 2010), but these results may be confounded by the background strain of mice, which shows substantial ARHL with minimal ARVL (see Mock et al., 2016).

Together these results indicate that oxidative stress likely plays a role in both ARHL and ARVL (Figure 4), although different antioxidant systems may be involved. Speculatively, these results suggest increased involvement of GPX and SOD in ARHL compared to increased involvement of catalase in ARVL, but more work is necessary. Unfortunately, the various mouse models that exist to examine age-related oxidative stress (reviewed in Hamilton et al., 2012) have not been leveraged to examine the contribution of oxidative stress to either ARHL or ARVL. The link between ARHL and other forms of acquired hearing loss (Yang et al., 2015) suggests the examination of models known to show increased susceptibility to other forms of acquired hearing loss to better understand ARHL. For example, GPX1 knockout mice (on a mixed C57BL/6 and 129/SVJ background) show increased susceptibility to noise-induced hearing loss (Ohlemiller et al., 2000), and, therefore, should be prioritized for investigation to identify the role of oxidative stress in ARHL and ARVL.

### Genetic Contributions

As multifactorial conditions, both ARHL and ARVL have underlying genetic contributions (Figure 4). Indeed, twin and family studies suggest that 25 to 75% of the risk for ARHL is due to heritable causes (see Yamasoba et al., 2013; Momi et al., 2015). Several genome-wide association studies (GWAS) and candidate gene studies have been performed to identify the specific genes contributing to ARHL (reviewed in Ahmadmehrabi et al., 2021). Recent work combining GWAS with other approaches (Nagtegaal et al., 2019; Wells et al., 2019; Kalra et al., 2020), including gene set enrichment analyses, transcriptomic and epigenomic data from the mouse cochlea, as well as immunohistochemistry in the mouse cochlea, implicate a role of at least some of these genes in metabolic, sensory, and neuronal functioning of the cochlea. The genetic contribution to most vestibular disorders, and specifically ARVL, remains largely unknown (Frejo et al., 2016; Gallego-Martinez et al., 2018), and GWAS have not yet been performed to identify the genetic mechanisms contributing to ARVL. Nevertheless, replicated candidate genes identified by GWAS of ARHL are expressed in the mouse utricle based on the SHIELD database (Shen et al., 2015), including (but not necessarily limited to) ILDR1 (Nagtegaal et al., 2019), a cell surface marker; ZNF318, a cell signaling molecule; NID2 a cell adhesion molecule; and ARHGEF28, a guanine nucleotide exchange factor with various functions. Additional prospective candidates for ARVL may be identified by applying a similar approach that used exome sequencing from individuals with ARHL to identify pathogenic variants in known (non-syndromic) deafness genes (Lewis et al., 2018; Boucher et al., 2020; de Bruijn et al., 2020). Finally, identification of the non-coding genetic elements (approximately 98% of the total human genome) contributing to ARHL and ARVL has only just begun despite the well-recognized contribution of non-coding genetic information to other diseases (reviewed in Esteller, 2011). Initial findings suggest overlapping candidates in both the cochlea and vestibular end organs (Schrauwen et al., 2016; Ushakov et al., 2017; Zhao et al., 2020). MicroRNAs (miRNAs), short non-coding RNA molecules that can specifically regulate gene expression by binding to complementary mRNA molecules, have been studied in the context of ARHL and show to regulate important biological processes in the aging cochlea (reviewed in Hu W. et al., 2018). These miRNAs are very stable and can be utilized as biomarkers and therapeutic targets (Pang et al., 2016; Hu W. et al., 2018).

### Overlapping and Interacting Mechanisms

The mechanisms highlighted above are closely linked and also interact with each other and other mechanisms associated with aging (Figure 4). Unchecked oxidative damage initiates inflammation that, in turn, produces ototoxic ROS and pro-inflammatory elements. Both oxidative stress and inflammation are linked to autophagy, mitophagy, and apoptosis as part of ARHL (reviewed in Wu J. et al., 2020). Oxidative stress is linked to accelerated senescence in a senescence mouse model (Hosokawa, 2002). Moreover, with aging, protein homeostasis – dubbed proteostasis – becomes less effective, so protein translation, chaperone-assisted protein folding, and protein degradation pathways become less accurate (reviewed in Kaushik and Cuervo, 2015). Along with faulty protein production and modification, inefficient removal of cellular waste products activates inflammatory pathways and results in cellular damage (Pizzino et al., 2017). Proteostasis is essential during cochlear development (Freeman et al., 2019) and is worthy of further investigation as a contributing factor to ARHL and ARVL. Dysregulation of proteostasis may give rise to altered ion and water homeostatic mechanisms, which have been indicated in dysfunction of the inner ear generally (Trune, 2010) and ARHL specifically (Peixoto Pinheiro et al., 2020). Intriguing work examining ARHL in the fruit fly indicates that the manipulation of evolutionary conserved transcription-factor homeostatic master regulators in the fly’s auditory neurons altered (accelerated or protected) the ARHL phenotype (Keder et al., 2020). Allelic variants of various genes can influence both the susceptibility to dysregulation of these various pathways as well as the susceptibility to functional and structural damage exerted by these pathways. Finally, potentially modifiable factors can also influence these mechanisms. Diet (Someya et al., 2010) and exercise (Han et al., 2016) influence ARHL, and exposure to ototoxins (Yang et al., 2015) and noise (Stewart et al., 2020) influence both acquired hearing loss and vestibular dysfunction (Figure 4).

Mechanisms, like cellular senescence, and structures, like mitochondria, may serves as common points of intersection or even central regulators in these interacting mechanisms. A senescence-accelerated mouse model (SAMP8 mice) shows accelerated ARHL associated with indicators of oxidative stress, inflammation, altered proteostasis, and apoptosis (Menardo et al., 2012), consistent with a role for cellular senescence as an overarching regulator of ARHL. Mitochondria, which are important for metabolism, redox homeostasis, signaling, and regulation of programmed cell death, are known to be involved in ARHL (Crawley and Keithley, 2011; Fujimoto and Yamasoba, 2014). Since comparatively less is known about the molecular mechanisms involved in ARVL, these common points of intersection may be especially fruitful avenues of investigation to identify mechanisms underlying ARVL. Interaction of multiple mechanisms may be good news for strategies to diagnosis and mitigate the effects of ARHL and ARVL, providing multiple biomarkers relevant for improved diagnosis and allowing multiple points of intervention to mitigate the effects of ARHL and ARVL.

Finally, additional insights into the mechanisms interrelating age-related pathology in the cochlea and vestibular end organs come from the long-recognized oto- and nephrotoxicity of some drugs, especially aminoglycoside antibiotics but also chemotherapeutics like cisplatin (Kros and Steyger, 2019). Like both ARHL and ARVL, ototoxicity is associated with cochlear and vestibular hair cell and synapse loss that progresses from high frequency and central/strial regions to low frequency and peripheral/extrastriolar regions of the inner ear sensory epithelia (e.g., Greguske et al., 2021). The link between oto- and nephrotoxicity appears to result from similarities between epithelial transport in the inner ear and kidney (Torban and Goodyer, 2009). Focusing on the inner ear, aminoglycosides quickly accumulate in the endolymph, presumably via capillaries of the stria vascularis. Various suspected routes, including endocytosis and transport through ion channels, lead to accumulation of aminoglycosides in the hair cells (Huth et al., 2011). Once in the hair cells, aminoglycosides lead to the formation of ROS and ultimately trigger apoptosis (Selimoglu, 2007). Various factors increase the risk of aminoglycoside ototoxicity, but genetic predisposition is one of the main determinants of susceptibility (Huth et al., 2011). These genetic mutations appear to affect mitochondrial function and to exacerbate cochleotoxicity preferentially. Together these and other findings suggest that genetic susceptibility to aminoglycoside ototoxicity arises from reduced mitochondrial function and, consequently, reduced strial function. There are still many discrepancies in the current understanding of aminoglycoside ototoxicity and the links shared with nephrotoxicity. Nevertheless, current insights suggest that altered transport mechanisms, oxidative stress, and genetic mutations, especially those affecting mitochondrial function, may be mechanisms underlying both ototoxicity and ARHL and ARVL and may be the mechanistic links connecting ARHL, ARVL and other comorbidities. More insights into these specific mechanisms are worthy of further investigation.

## Mechanisms Underlying Age-Related Neurodegeneration Inform Age-Related Pathology in the Cochlea and Vestibular End Organs

The central auditory and vestibular systems also show changes associated with age-related decline in hearing and balance perception that may occur as part of brain aging and/or be driven by age-related changes in the cochlea and vestibular end organs. These changes include both anatomical and functional alterations of the brain (reviewed in Arshad and Seemungal, 2016; Sardone et al., 2019; Slade et al., 2020) and are briefly summarized here. Anatomically, ARHL has been associated with shrinking of the total brain volume (Lin et al., 2014) as well as reduced gray matter volume specifically in the auditory cortex (Eckert et al., 2012) and right temporal lobe (Lin et al., 2014). Brain imaging using functional magnetic resonance imaging (fMRI) has shown that ARHL is associated with functional changes in auditory and non-auditory brain regions as well as functional connectivity in networks important for auditory perception (reviewed in Slade et al., 2020). The landmark cross-sectional study of Lin and colleagues revealed a higher risk of dementia with ARHL (Lin et al., 2011a). Subsequent cross-sectional and a few longitudinal studies confirm an association between ARHL and cognitive impairment and dementia (Livingston et al., 2017; Loughrey et al., 2018). However, more recent work indicates that the association between hearing loss and accelerated cognitive decline was non-significant after additionally adjusting for faster cognitive decline at older ages, which was not done in previous studies (Croll et al., 2021). More evidence is necessary to substantiate the role of hearing loss as a modifiable risk factor for cognitive decline.

Various age-related anatomical and functional alterations have been reported in the central vestibular system. An age-related decrease in volume (Lopez et al., 1997) and neuronal density (Lopez et al., 1997; Tang et al., 2001) and increase in neurons with lipofuscin deposits (Lopez et al., 1997; Alvarez et al., 2000) have been reported in the vestibular nuclear complex. Various age-related changes in the cerebellum have also been reported, including reduced volume of the cerebellar vermis (Torvik et al., 1986; Luft et al., 1999) and reduced global white matter of the cerebellum (Andersen et al., 2003). Age-related decline in functional connectivity of the vestibular cortical network has also been observed using galvanic vestibular stimulation (GVS), which bypasses the peripheral vestibular system and directly stimulates the vestibular nerve. An inverse U-shape in vestibular functional response, measured by torsional eye movements in response to GVS, was found (Jahn et al., 2003). In this study, the authors speculate that central processing compensates for the reduction in vestibular hair cells and other peripheral changes by becoming hypersensitive, but, after the sixth decade, becomes insufficient. Recent work indicates that ARVL, like ARHL, is associated with the increased likelihood and rate of cognitive decline in the aging (Semenov et al., 2016; Dobbels et al., 2019).

How ARHL and ARVL are linked to cognitive impairment is not revealed in association studies, but several conceptual models have been proposed and evaluated for ARHL (e.g., Davis et al., 2016; Chern and Golub, 2019; Griffiths et al., 2020; Slade et al., 2020; Nadhimi and Llano, 2021) and are useful when considering ARVL (Smith, 2021). One possibility is that there are common mechanisms, such as vascular and/or neural pathology, that independently give rise to ARHL/ARVL and cognitive impairment. Another possibility is that ARHL/ARVL indeed causes cognitive impairment. The underlying mechanism is not known but reduced input from the cochlea and vestibular end organs may lead to (1) an impoverished or distorted sensory environment (which may contribute to or be exacerbated by social isolation), (2) reallocation of cognitive resources for listening tasks that reduces resources available for other cognitive tasks, and (3) functional and anatomical changes in the brain. While these underlying mechanisms have been specifically proposed for ARHL, loss of peripheral vestibular input would also be expected to have enormous consequences for cognition given the vast network in the cerebral cortex activated by vestibular stimulation (Lobel et al., 1998; Brandt and Dieterich, 1999; Lopez et al., 2012). Importantly, these underlying mechanisms may interact with one another in complex ways (Johnson et al., 2020), and recent works suggest that distinct mechanisms may be responsible, with different types of dementia associated with different patterns of hearing loss (Jung et al., 2021).

Various neurodegenerative disorders associated with cognitive decline and dementia frequently present not only with alterations in the central auditory and vestibular pathways but also with and peripheral auditory and/or vestibular impairment (Hardy et al., 2016; Cronin et al., 2017). Thus, the mechanisms underlying these disorders provide insights into potentially shared mechanisms underlying age-related auditory and vestibular end organ dysfunction. Among the most common neurodegenerative disorders associated with age-related dementia concomitant with auditory and vestibular impairment are Alzheimer’s and Parkinson’s diseases. Huntington’s disease is an inherited neurodegenerative disease that presents in middle age and is also associated with hearing impairment.

Alzheimer’s disease is the most common cause of dementia in the elderly and is frequently associated with ARHL (Shen et al., 2018). Inflammation-associated neurovascular decline in the blood-brain barrier and blood-labyrinth barrier has been offered as the common cause of both Alzheimer’s disease and ARHL (Shityakov et al., 2021). Moreover, cochlear pathology, including loss of auditory spiral ganglion neurons and sensory hair cells, abnormal deposition of amyloid β protein, and overexpression of tau protein in cochlea hair cells have been reported in mouse models of Alzheimer’s disease and implicated in loss of cochlear function (reviewed in Shen et al., 2018). Examination of peripheral vestibular pathology in mouse models of Alzheimer’s disease remains to be done. In humans, the link between Alzheimer’s disease and peripheral vestibular dysfunction is unclear, with some studies identifying a link (e.g., Harun et al., 2016) and others finding no association. A recent systematic review examining the association between either Alzheimer’s disease and mild cognitive impairment found no correlation between cognitive decline and semicircular canal function using vHIT, rotatory chair testing, and caloric irrigation (Bosmans et al., 2021). However, the authors of this study suggest cautious interpretation of these findings given the limited number of available studies (seven studies with a combined total of 235 subjects with impaired cognition) and the large heterogeneity in outcome measures. Nevertheless, these results tentatively implicate a role for neurovascular decline and amyloid beta aggregation, which results in oxidative stress, inflammation, and ultimately neuronal cell death (Querfurth and LaFerla, 2010; Heneka et al., 2015), as mechanisms interrelating peripheral and central dysfunction associated with Alzheimer’s disease.

Like Alzheimer’s disease, Parkinson’s disease and the related Lewy body disease are also associated with cognitive impairment in middle to late life and both peripheral auditory and vestibular impairment (Hardy et al., 2016; Jafari et al., 2020; Johnson et al., 2020). Multiple studies report increased audiometric hearing thresholds (after adjustment for the effect of ARHL) in patients with Parkinson’s disease and reduced otoacoustic emission (OAE) amplitudes (reviewed in Jafari et al., 2020; De Groote et al., 2021). Even asymptomatic hearing impairment frequently occurs in early-onset Parkinson’s Disease (Shetty et al., 2019). The link between Parkinson’s disease and peripheral vestibular impairment is less clear but has been documented using both caloric tests and VEMPs (Smith, 2018). Previous work in mice found reduced α-synuclein and synaptophysin expression in the cochlea, and especially medial olivocochlear efferent terminals contacting the outer hair cells, in C57 mice (which show accelerated age-related hearing loss) compared to CBA mice (which show little age-related hearing loss), suggesting a link between α-synuclein and susceptibility to age-related cochlear pathology (Park et al., 2011). α-synuclein is a neuronal protein that regulates synaptic vesicle trafficking and neurotransmitter release (Burre et al., 2018). Insoluble fibrils of α-synuclein aggregates are characteristically seen in Parkinson’s disease and other neurodegenerative disorders such as dementia with Lewy bodies, but the mechanisms by which α-synuclein acts in neurodegeneration, as well as its normal cellular function, are unknown. The combination of findings described here tentatively suggest that Parkinson’s disease and peripheral auditory impairment may result from altered synaptic transmission due to altered α-synuclein expression both centrally and peripherally. In addition to altered synaptic transmission, mitochondrial dysfunction has also been proposed to interrelate Parkinson’s disease and at least ARHL (Jafari et al., 2020).

Huntington’s disease is associated with cognitive decline and at least central auditory impairment (Hardy et al., 2016). The link between Huntington’s disease and peripheral auditory impairment is less clear. One study found that patients with Huntington’s disease had elevated auditory thresholds (based on PTA and ABR) compared to healthy controls (Lin et al., 2011c). This study also reported elevated auditory thresholds (based on ABR) in a mouse model of Huntington’s disease. Additional findings from this study implicated mutant huntingtin protein expression in the organ of Corti and reduced expression of brain creatine kinase in the cochlea of this mouse model of Huntington’s disease. Another study, however, found central but not peripheral auditory impairment in patients with Huntington’s disease (Profant et al., 2017). Vestibular impairment has not been directly investigated in patients with Huntington’s disease or mouse models of Huntington’s disease. Although the exact pathophysiology underlying Huntington’s disease is not known, aggregates of mutant huntingtin, which form inclusion bodies, may disrupt neuronal homeostasis (Jimenez-Sanchez et al., 2017) and, in this way, disrupt function of both the brain and cochlea.

More generally, markers of inflammation are associated with a variety of neurodegenerative disorders (Kinney et al., 2018) and linked to peripheral auditory impairment, including Neuro-Behçet’s syndrome, antiphospholipid syndrome, multiple sclerosis, and neurological sarcoidosis (Hardy et al., 2016). Each of these neurodegenerative disorders has also been linked to vestibular impairment (e.g., Gemignani et al., 1991; Colvin, 2006; Girasoli et al., 2018; MacMahon and El Refaie, 2021). Thus, inflammation likely interrelates age-related cognitive and peripheral auditory and vestibular impairment.

Identifying the complex interactions between age-related changes in the auditory and vestibular end organs, central auditory and vestibular pathways, and cognitive impairment is critical to discovering the most effective strategies to prevent, stall, and perhaps even reverse ARHL, ARVL, and related comorbidities. Loss of peripheral auditory (and likely also vestibular) input is itself and regardless of aging a potent trigger of changes in brain structure and function and involves various synaptic mechanisms, ascending and descending auditory pathways, and neuromodulatory circuits (Persic et al., 2020). Thus, age-related loss of cochlea and vestibular end organ function may be the primary drivers of changes in the central auditory and vestibular pathways and various brain regions involved in age-related comorbidities, including cognitive decline. Thus, proactive treatment of ARHL and ARVL targeted at peripheral restoration is especially strategic and also associated with low risk and added health benefits (Ferguson et al., 2017). Moreover, understanding the peripheral pathology underlying ARHL and ARVL is crucial regardless of whether there is a common, causal, or even unique mechanisms linking ARHL/ARVL and various comorbidities. Insights will allow identification of the key common pathologies and/or lead to the development of methods that better preserve or more fully restore peripheral auditory and vestibular input, which may ultimately be necessary to prevent the host of comorbidities associated with age-related decline of the cochlea and vestibular end organs.

## Conclusion

Both ARHL and ARVL are prevalent conditions associated with changes in the structure and function of the cochlea and vestibular end organs. A careful comparison of these changes reveals that the patterns of pathophysiology show similarities but also differences both between the cochlea and vestibular end organs and among the vestibular end organs. This review uncovers gaps in the current research landscape and identifies specific, high impact research objectives that should be prioritized. Specifically:

• Assessments of inner ear function in both humans and animal models using a combination of tests, which provide complementary rather than redundant information, are needed to identify and examine correlations between ARHL and ARVL.
• Epidemiological studies of the prevalence of ARVL and associated risk factors, including genetic risk factors, for ARVL that utilize standardized, quantitative, clinical assessments of vestibular end organ function are needed.
• Analogous and robust assessments of peripheral vestibular function in humans and animal models need to be developed and applied.
• Animal models, which have been developed to examine other age-related conditions, should be better leveraged to identify the mechanisms contributing to ARHL and especially ARVL. The background strain of mouse models should be considered carefully since there is considerable strain-dependent variation in the susceptibility to age-related hearing loss and audiogenic seizures.
• The relationship between age-related changes in the peripheral and central auditory and vestibular systems need to be examined, especially when considering the complex, multisystem complications, like falls, depression, and cognitive decline, shared by these conditions.

Addressing these gaps will require integrated investigation of the auditory and vestibular structures of the inner ear using both clinical and animal models. Ultimately, because both ARHL and ARVL are associated with and likely mechanistically linked to other age-related conditions, treatments to prevent ARHL and ARVL are essential to strategies promoting healthy aging overall.

## Author Contributions

All authors contributed to data collection, analysis and interpretation of data, manuscript preparation, reviewed, and approved the final version of the manuscript.

## Conflict of Interest

The authors declare that the research was conducted in the absence of any commercial or financial relationships that could be construed as a potential conflict of interest.

## Publisher’s Note

All claims expressed in this article are solely those of the authors and do not necessarily represent those of their affiliated organizations, or those of the publisher, the editors and the reviewers. Any product that may be evaluated in this article, or claim that may be made by its manufacturer, is not guaranteed or endorsed by the publisher.
